# Natural Products Chemistry: Advances in Synthetic, Analytical and Bioactivity Studies, Volume II

**DOI:** 10.3390/molecules31020374

**Published:** 2026-01-20

**Authors:** Giovanni Ribaudo

**Affiliations:** Department of Molecular and Translational Medicine, University of Brescia, Viale Europa 11, 25123 Brescia, Italy; giovanni.ribaudo@unibs.it

The idea of collecting novel contributions relating to the chemistry of natural compounds in this Special Issue stemmed from the success of the first edition of the collection entitled “Natural Products Chemistry: Advances in Synthetic, Analytical and Bioactivity Studies”, which was published in *Molecules* in 2023 [1].

The field of chemical sciences, and of medicinal chemistry in particular, has always been strongly related to the world of natural products. Small organic compounds of natural origin are chemically diverse molecules, and they are characterized by a variety of scaffolds and functional groups. Moreover, chirality represents an additional feature [2].

The study of natural compounds is intriguing because of their potential applications, but it also carries several challenges from the point of view of their extraction and analytical characterization. At the same time, organic chemists put their best effort in the efficient synthesis and derivatization and optimization of natural compounds to produce optimized analogs, thus unleashing the potential of the semi-synthetic derivatives [3]. Flavonoids, alkaloids and terpenes are only some of the chemical classes that attract the interest of the medicinal chemist for the identification and development of novel therapeutic options [4,5]. Eventually, it must be considered that currently used theoretical drug discovery tools allow us to rationalize and translate into modern medicinal chemistry the traditional uses of nature-inspired molecules [6,7,8]. Indeed, medicinal chemistry is one of the fields of chemical sciences in which natural compounds traditionally find wide application. Such molecules are investigated for their anticancer roles through peculiar mechanisms [9], as antibacterial agents [10] and with the aim of developing novel tools against neurodegeneration [11,12]. Nevertheless, natural compounds are the focus of many other fields [13]. In this connection, food chemistry [14], engineering and material sciences represent only few examples [15,16].

This Special Issue aimed at collecting research papers and review articles related to the different aspects of the chemistry of natural compounds, including extraction, structural elucidation, synthesis and biological evaluation of natural, semi-synthetic derivatives and nature-inspired molecules. Particular attention was also dedicated to compounds of pharmaceutical interest. As a result, between 2024 and 2025, eight articles were published in this Special Issue, testifying the strong interest of the scientific community towards the chemistry of natural compounds under different perspectives. In particular, the Special Issue collected four research articles and four reviews. Moreover, it must be noted that this volume showed an international reach, as authors across the globe submitted their contributions. Indeed, corresponding authors from China, Ecuador, Italy, Japan, Poland, Saudi Arabia, South Africa, and USA, along with their collaborators from many other countries such as Egypt, Palestine, and Ukraine participated to this Special Issue with their research.

In further detail, the research papers are concerned with the following: Dushna and colleagues synthesized N-oxide derivatives of alkaloids and characterized them through electrochemical studies. In the context of organic synthesis, Webber and Russu described trimethylenemethane cyclopentyl-annulations as a strategy to obtain a functionalized angular triquinane skeleton. Ayoup and colleagues reported the synthesis, characterization and antiproliferative activity of fluorinated isoflavones. Hu and colleagues analyzed the constituents, of medicinal interest, of *Andrographis paniculata* (Burm. f.).

Concerning review articles, Khwaza and Aderibigbe focused their contribution on the pharmacological properties of derivatives of ursolic acid. Adriana Monserrath Orellana-Paucar reviewed the therapeutic properties of turmeric essential oil components. In their contribution, Kato-Noguchi and Kato reviewed the defense molecules of the invasive plant species *Ageratum conyzoides*, while Antonio Evidente provided an overview of α-pyrones as phytotoxins produced by plant pathogen fungi.

The contributions collected in the Special Issue are listed below, following the order presented above.

- Dushna, O.; Dubenska, L.; Gawor, A.; Karasińki, J.; Barabash, O.; Ostapiuk, Y.; Blazheyevskiy, M.; Bulska, E. Structural Characterization and Electrochemical Studies of Selected Alkaloid N-Oxides. *Molecules* **2024**, *29*, 2721. https://doi.org/10.3390/molecules29122721.
- Webber, S.M.; Russu, W.A. Investigation of Trimethylenemethane Cyclopentyl-Annulations as a Strategy to Obtain a Functionalized Angular Triquinane Skeleton. *Molecules* **2024**, *29*, 5358. https://doi.org/10.3390/molecules29225358.
- Ayoup, M.S.; Daqa, M.; Salama, Y.; Hazzam, R.; Hawsawi, M.B.; Soliman, S.M.; Al-Maharik, N. Efficient Consecutive Synthesis of Fluorinated Isoflavone Analogs, X-Ray Structures, Hirshfeld Analysis, and Anticancer Activity Assessment. *Molecules* **2025**, *30*, 795. https://doi.org/10.3390/molecules30040795.
- Hu, E.; Cheng, R.; Liu, A.; Wang, Y.; Long, H.; Hou, J.; Wang, D.; Wu, W.; Wu, X. Metabolomic Profiles and Differential Constituents of Andrographis paniculata (Burm. f.) in Different Growth Stages and Parts. *Molecules* **2025**, *30*, 1490. https://doi.org/10.3390/molecules30071490.
- Khwaza, V.; Aderibigbe, B.A. Potential Pharmacological Properties of Triterpene Derivatives of Ursolic Acid. *Molecules* **2024**, *29*, 3884. https://doi.org/10.3390/molecules29163884.
- Orellana-Paucar, A. Turmeric Essential Oil Constituents as Potential Drug Candidates: A Comprehensive Overview of Their Individual Bioactivities. *Molecules* **2024**, *29*, 4210; https://doi.org/10.3390/molecules29174210.
- Kato-Noguchi, H.; Kato, M. Defense Molecules of the Invasive Plant Species Ageratum conyzoides. *Molecules* **2024**, *29*, 4673. https://doi.org/10.3390/molecules29194673.
- Evidente, A. An Overview of α-Pyrones as Phytotoxins Produced by Plant Pathogen Fungi. *Molecules* **2025**, *30*, 2813. https://doi.org/10.3390/molecules30132813.

In conclusion, the success of this Special Issue once again testifies the interest of the scientific community on the field of natural compounds, and prompts further research in the field.
